# How sample size influences research outcomes

**DOI:** 10.1590/2176-9451.19.4.027-029.ebo

**Published:** 2014

**Authors:** Jorge Faber, Lilian Martins Fonseca

**Affiliations:** 1 Adjunct professor, Department of Orthodontics, University of Brasília.; 2 Invited Professor, Department of Orthodontics, University of Brasília.

**Keywords:** Sample calculation, Sample size, Clinical trial, Methodology, Scientific evidence

## Abstract

Sample size calculation is part of the early stages of conducting an epidemiological, clinical or lab study. In preparing a scientific paper, there are ethical and methodological indications for its use. Two investigations conducted with the same methodology and achieving equivalent results, but different only in terms of sample size, may point the researcher in different directions when it comes to making clinical decisions. Therefore, ideally, samples should not be small and, contrary to what one might think, should not be excessive. The aim of this paper is to discuss in clinical language the main implications of the sample size when interpreting a study.

## Abstract (pt)

O cálculo amostral faz parte dos estágios iniciais de realização de um estudo epidemiológico, clínico ou laboratorial. Há indicações éticas e metodológicas para o seu emprego na elaboração de um trabalho científico. Duas pesquisas, realizadas com a mesma metodologia obtendo resultados equivalentes, e que diferem apenas no tamanho da amostra, podem apontar para diferentes direções no processo de tomada de decisão clínica. Portanto, as amostras estudadas idealmente não devem ser pequenas e, ao contrário do que pode-se pensar, não devem ser excessivas. O objetivo desse artigo é discutir, numa linguagem clínica, as principais implicações do tamanho das amostras na interpretação de um estudo.

In recent years a growing concern has overwhelmed the scientific community in the healthcare area: Sample size calculation. Although at first blush it may seem like an overriding concern over methodological issues, notably to clinicians, such concern is utterly justifiable. This issue is of paramount importance.

Samples should not be either too big or too small since both have limitations that can compromise the conclusions drawn from the studies. Too small a sample may prevent the findings from being extrapolated, whereas too large a sample may amplify the detection of differences, emphasizing statistical differences that are not clinically relevant.^1^ We will discuss in this article the major impacts of sample size on orthodontic studies.

## FACTORS THAT AFFECT SAMPLE SIZE

The purpose of estimating the appropriate sample size is to produce studies capable of detecting clinically relevant differences. Bearing this point in mind, there are different formulas to calculate sample size.^2,3^ These formulas comprise several aspects which are listed below. Most sample size calculators available on the web have limited validity because they use a single formula - which is usually not divulged - to generate sample sizes for the studies.

The first aspect is the type of variable being studied. For example, it should be determined if the variable is categorical like the Angle classification (Class I, II or III), or continuous like the length of the dental arch (usually measured in millimeters).

It is then necessary to determine the relationship between the groups that will be evaluated and the statistical analysis that will be employed. Are we going to evaluate groups that are independent, i.e., the measurements of one group do not influence the other? Are they dependent groups like the measurements taken before and after treatment? Are we going to use a split-mouth design, whereby treatment is performed on one quadrant and a different therapy on another quadrant? Will we be using t-test or chi-square test? All these questions lead to different sample size calculation formulas.

Subsequently, we have to answer the question concerning which results we envisage if a standard treatment is performed. What is the mean value or the expected ratio? The answer to this question is usually obtained from the literature or by means of pilot studies.

It is also important to determine what is the smallest magnitude of the effect and the extent to which it is clinically relevant. For example, how many degrees of difference in the ANB angle can be considered relevant? It is vital that we address this issue. The smaller the difference that we wish to identify, the greater the number of cases in a study. If researchers wish to detect a difference as small as 0.1° in an ANB angle, they will probably need thousands of patients in their study. If this value rises to 1°, the number of cases required falls drastically.

Finally, it is essential that the researcher determine the level of significance and the type II error, which is the probability of not rejecting the null hypothesis, although the hypothesis is actually false, which the study will accept as reasonable.

With this information in hand, we will apply the appropriate formula according to the study design in question, and determine the sample size. Today, this calculation is typically carried out with the aid of a computer program. For example, Pocock's formula^2^ for continuous variables is frequently used in our specialty. It is used in studies where one wishes to examine the difference between data means with normal distribution and equal-size, independent groups.

## PROBLEMS WITH VERY SMALL SAMPLES

Try to envision the following scenario. A researcher conducts a study on patients who are being treated with a new device which although very uncomfortable has the potential to improve treatment of Class II malocclusions. The researcher wishes to compare the new functional device with the Herbst appliance. Patients will be randomly assigned to each group. The researcher is not aware, but we are, that s/he needs 60 subjects (30 patients in each group) to ensure sufficient power to be able to extrapolate the statistical analysis results to the overall population. In other words, so that we can feel confident that these results will serve as a parameter on which to base the proposed treatment. Furthermore, we also know, although the researcher does not, that this new therapy is less effective than the traditional method.

However, the researcher used only 15 patients in each group. The results of the study showed that the new device is inferior to conventional treatment. What are the implications?

The first is that using a sample smaller than the ideal increases the chance of assuming as true a false premise. Thus, chances are that the proposed device has no disadvantage compared to traditional therapy. Furthermore, it is assumed that people were subjected to a study, and had to undergo in vain all additional suffering associated with the therapy, given that the goals of the study were not achieved. In addition, financial and time resources were squandered since ultimately it will contribute absolutely nothing to improve clinical practice or quality of life. The situation becomes even worse if the research involves public funding: A total waste of taxpayer money.

## PROBLEMS WITH VERY LARGE SAMPLES

There is a widespread belief that large samples are ideal for research or statistical analysis. However, this is not always true. Using the above example as a case study, very large samples that exceed the value estimated by sample size calculation present different hurdles.

The first is ethical. Should a study be performed with more patients than necessary? This means that more people than needed are exposed to the new therapy. Potentially, this implies increased hassle and risk. Obviously the problem is compounded if the new protocol is inferior to the traditional method: More patients are involved in a new, uncomfortable therapy that yields inferior results.

The second obstacle is that the use of a larger number of cases can also involve more financial and human resources than necessary to obtain the desired response.

In addition to these factors, there is another noteworthy issue that has to do with statistics. Statistical tests were developed to handle samples, not populations. When numerous cases are included in the statistics, analysis power is substantially increased. This implies an exaggerated tendency to reject null hypotheses with clinically negligible differences. What is insignificant becomes significant. Thus, a potential statistically significant difference in the ANB angle of 0.1° between the groups cited in the previous example would obviously produce no clinical difference in the effects of wearing an appliance.

When very large samples are available in a retrospective study, the researcher needs first to collect subsamples randomly, and only then perform the statistical test. If it is a prospective study, the researcher should collect only what is necessary, and include a few more individuals to compensate for subjects that leave the study.

## CONCLUSIONS

In designing a study, sample size calculation is important for methodological and ethical reasons, as well as for reasons of human and financial resources. When reading an article, the reader should be on the alert to ascertain that the study they are reading was subjected to sample size calculation. In the absence of this calculation, the findings of the study should be interpreted with caution.

An appropriate sample renders the research more efficient: Data generated are reliable, resource investment is as limited as possible, while conforming to ethical principles. The use of sample size calculation directly influences research findings. Very small samples undermine the internal and external validity of a study. Very large samples tend to transform small differences into statistically significant differences - even when they are clinically insignificant. As a result, both researchers and clinicians are misguided, which may lead to failure in treatment decisions.

## References

[1] Altman DG (1991). Practical Statistics for Medical Research.

[2] Pandis N, Polychronopoulou A, Eliades T (2011). Sample size estimation: an overview with applications to orthodontic
clinical trial designs. Am J Orthod Dentofacial Orthop..

[3] Hajian-Tilaki K (2014). Sample size estimation in diagnostic test studies of biomedical
informatics. J Biomed Inform..

